# Identification of dynamic glucocorticoid-induced methylation changes at the *FKBP5* locus

**DOI:** 10.1186/s13148-019-0682-5

**Published:** 2019-05-23

**Authors:** Tobias Wiechmann, Simone Röh, Susann Sauer, Darina Czamara, Janine Arloth, Maik Ködel, Madita Beintner, Lisanne Knop, Andreas Menke, Elisabeth B. Binder, Nadine Provençal

**Affiliations:** 1 Department of Translational Research in Psychiatry, Max Planck Institute of Psychiatry, Kraepelinstr. 2-10, 80804 Munich, Germany; 2 Department of Psychiatry, Psychosomatics and Psychotherapy, University Hospital of Wuerzburg, Wuerzburg, Germany; 3 Comprehensive Heart Failure Center, University Hospital of Wuerzburg, Wuerzburg, Germany; 4 Department of Psychiatry and Behavioral Sciences, Emory University Medical School, Atlanta, GA USA; 5 Faculty of Health Sciences, Simon Fraser University, 8888 University Drive, Burnaby, BC V5A 1S6 Canada; 6 BC Children’s Hospital Research Institute, Vancouver, BC Canada; 7 Institute of Computational Biology, Helmholtz Zentrum München, Neuherberg, Germany

**Keywords:** DNA methylation, *FKBP5*, Glucocorticoid receptor, Early-life stress, Targeted bisulfite sequencing, Dexamethasone

## Abstract

**Background:**

Epigenetic mechanisms may play a major role in the biological embedding of early-life stress (ELS). One proposed mechanism is that glucocorticoid (GC) release following ELS exposure induces long-lasting alterations in DNA methylation (DNAm) of important regulatory genes of the stress response. Here, we investigate the dynamics of GC-dependent methylation changes in key regulatory regions of the *FKBP5* locus in which ELS-associated DNAm changes have been reported.

**Results:**

We repeatedly measured DNAm in human peripheral blood samples from 2 independent cohorts exposed to the GC agonist dexamethasone (DEX) using a targeted bisulfite sequencing approach, complemented by data from Illumina 450K arrays. We detected differentially methylated CpGs in enhancers co-localizing with GC receptor binding sites after acute DEX treatment (1 h, 3 h, 6 h), which returned to baseline levels within 23 h. These changes withstood correction for immune cell count differences. While we observed main effects of sex, age, body mass index, smoking, and depression symptoms on *FKBP5* methylation levels, only the functional *FKBP5* SNP (rs1360780) moderated the dynamic changes following DEX. This genotype effect was observed in both cohorts and included sites previously shown to be associated with ELS.

**Conclusion:**

Our study highlights that DNAm levels within regulatory regions of the *FKBP5* locus show dynamic changes following a GC challenge and suggest that factors influencing the dynamics of this regulation may contribute to the previously reported alterations in DNAm associated with current and past ELS exposure.

**Electronic supplementary material:**

The online version of this article (10.1186/s13148-019-0682-5) contains supplementary material, which is available to authorized users.

## Background

Epidemiological studies indicate a combined contribution of genetic and environmental factors in the risk for psychiatric diseases, which converge to alter gene regulation and consequently cell function [1]. Evidence suggests that epigenetic mechanisms play a major role in embedding environmental risk, including early-life adversity, but our understanding of the underlying mechanisms is limited. Epigenetic mechanisms encompass post-translational modifications of histone proteins and chemical modifications of single nucleotides (most commonly in the form of methylation at cytosine guanine dinucleotides (CpGs)), which alter chromatin structure, and thus accessibility of the DNA to transcriptional regulators.

Even DNA methylation (DNAm), a stable chemical modification, undergoes highly dynamic regulation in post-mitotic cells. This property makes DNAm a suitable molecular mechanism to encode the impact of environmental cues in post-mitotic tissue [2, 3]. A mechanism that likely contributes to such dynamic, environmentally triggered DNAm changes is transcription factor-mediated DNA demethylation [4]. One example is local demethylation of glucocorticoid response elements (GREs) following activation of the glucocorticoid receptor (GR), a nuclear transcription factor [5]. The GR is activated by the glucocorticoid (GC) cortisol, a major mediator of the stress response.

Stress, especially in the form of early adverse life trauma, is a major environmental risk factor for psychiatric disorders [1, 6, 7]. Excessive GC release after stress exposure may induce long-lasting DNAm changes, thereby contributing to the biological embedding of risk trajectories. The mechanism of GR-induced local demethylation is not fully understood, but activation of DNA repair machinery is implicated. Demethylation of GREs facilitates the transcriptional effects of the GR on the target gene [8, 9].

*FKBP5* is a stress-responsive gene and co-chaperone protein of GR. Increased activation of this gene by genetic or epigenetic factors has been repeatedly associated with increased stress-sensitivity and risk for psychiatric disorders in both animal and human studies (see [10, 11] for review). We have previously reported on GR-sensitive CpGs in GREs of the *FKBP5* locus. These are located in a functional GRE in intron 7 of the gene. Chromatin conformation capture experiments confirmed an interaction of this intronic enhancer with the transcription start site (TSS) of *FKBP5.* Reporter gene assays also demonstrated that higher DNAm of this enhancer region was associated with lower transcriptional activation of *FKBP5* by GCs [12]. Relative reduction of DNAm in this region has been reported both in peripheral blood and buccal cells of adults as well as children exposed to childhood trauma and in a hippocampal neuronal progenitor cell (HPC) line following exposure to GCs [12–16]. Changes in DNAm following exposure to child abuse seemed to be accentuated in individuals carrying the minor allele of a functional genetic variant in this locus (rs1360780). This variant, located in close proximity to a GRE in intron 2, alters the 3D conformation of the locus. The minor allele generates a TATA-box binding site which allows binding of this intron enhancer to the transcription start site. This is also associated with higher *FKBP5* induction following GR activation. We and others have shown that increased *FKBP5* leads to reduced GR sensitivity and impaired negative feedback regulation of the stress hormone axis [17, 18]. In fact, minor allele carriers have repeatedly been shown to have prolonged cortisol release following stress exposure [11]. Finally, this functional allele has consistently been shown to increase risk for a range of psychiatric disorders with exposure to early adversity [11, 19–21], suggesting that gene x environment interactions at the level of epigenetic regulation may contribute to disease risk.

These studies suggest that the CpGs associated with early trauma exposure may also be responsive to GCs and that increased GR activation with trauma may lead to DNAm changes of the sites. However, direct evidence for this is so far missing. Furthermore, due to the limitation of the previously used pyrosequencing-based DNAm assessment of these enhancers, only a small number of CpGs had been investigated. Transcriptional regulatory sites of *FKBP5* are distributed throughout the locus and include several upstream, downstream, and intronic enhancer regions with GREs [22] as well as CCCTC-binding factor (CTCF) sites in addition to the TSS. CTCF creates boundaries between topologically associating domains (TADs) in chromosomes, and within these domains, CTCF facilitates interactions between transcription regulatory sequences [23, 24]. The extent of GR-associated DNAm changes in different categories of regulatory elements within the FKBP5 locus has not yet been explored.

Here, we investigate the changes of DNAm following exposure to the selective GR agonist dexamethasone (DEX) in peripheral blood cells over 24 h and in relation to rs1360780 genotype, in two independent cohorts. DNAm levels were assessed using a high-accuracy methylation measurements via targeted bisulfite sequencing (HAM-TBS) approach [25], which extensively covers CpG sites located in the different categories of regulatory elements in the *FKBP5* locus. The changes are also compared to data generated by the widely used Illumina methylation arrays.

## Results

### DEX-induced dynamic changes at the *FKBP5* locus in human peripheral blood (study 1)

In order to test if GR activation is associated with changes in DNAm in vivo, we first analyzed serial blood samples from 19 subjects exposed to a single oral dose (1.5 mg) of DEX (see Table 1 for demographic details).

**Table 1:** Description of study 1 and 2 subjects

	Study 1	Study 2
Samples	19	89
Male	19	67
Female	0	22
rs1360780 genotype	CC = 6; CT = 6; TT = 7	CC = 50; CT = 30; TT = 9
Time points of blood draw after DEX	0 h, 1 h, 3 h, 6 h, 23 h	0 h, 3 h, 18–24 h
Age (mean ± SD)	25.4 ± 2.9	41.6 ± 14.0
BMI (mean ± SD)	N/A	25.1 ± 3.8
Smoking score (mean ± SD)	N/A	− 0.6 ± 4.9
Major depressive disorder	0	59 (M = 38; F = 21)

#### DEX-induced changes in ACTH, cortisol, and FKBP5 mRNA levels

**Fig. 1:**
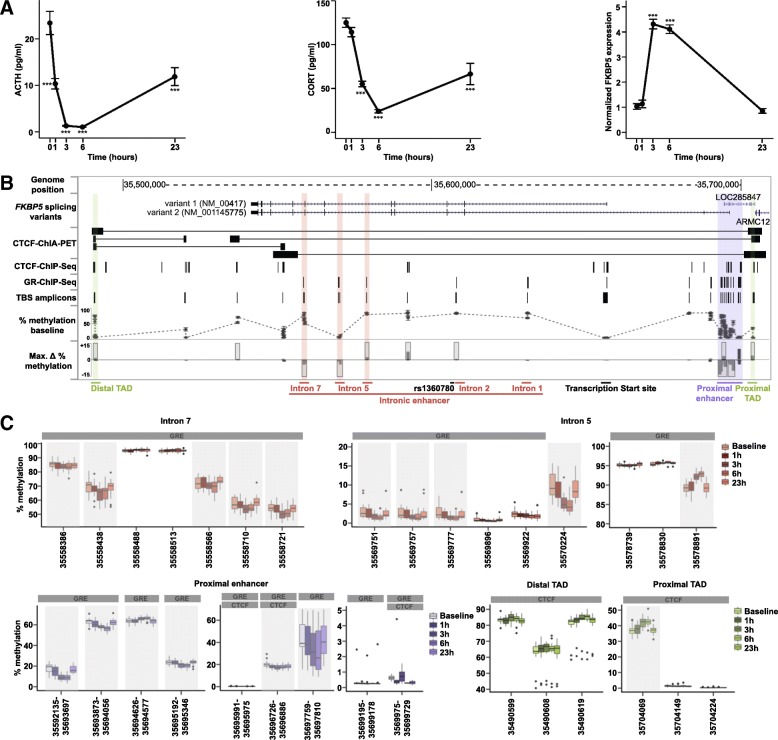
Dexamethasone (DEX)-induced transient changes in hormonal, *FKBP5* mRNA and methylation levels in blood. **a** Serum adrenocorticotropin (ACTH) and cortisol (CORT) levels as well as whole blood *FKBP5* mRNA levels after an oral dose of DEX in 19 healthy male subjects are shown. Peripheral blood was drawn just before administration of DEX (time = 0) as well as 1, 3, 6, and 23 h thereafter. The mean and SEM are presented for each time point. Linear mixed models showed a significant effect across time for ACTH (*p* value = 1.26e−23), CORT (*p* value = 1.18e−24), and *FKBP5* mRNA (*p* value < 2.2e−16) levels. *p* values of linear mixed models for each time point are indicated as follows: *≤ 0.05, **≤ 0.01, ***≤ 0.001. **b** Genome browser shot illustrating *FKBP5* regulatory elements and DEX-induced methylation changes across the locus (hg19/chr6:35487554-35718452). Genes, genes located within the locus; CTCF-ChIA-PET, locations of CTCF factor-mediated chromatin interactions determined by Chromatin Interaction Analysis with Paired-End Tag (ChIA-PET) data extracted from lymphoblastoid cell line (GM12878, [26]). Chromatin interactions are represented by PET blocks connected with an horizontal line. CTCF-ChIP-seq and GR-Chip-seq, regions of transcription factor binding derived from chromatin immunoprecipitation (ChIP) experiments in multiple cell lines from the ENCODE project; TBS amplicons, locations of targeted bisulfite sequencing (TBS) amplicons; % methylation baseline, methylation levels across TBS amplicons at baseline; Max. ∆ % methylation, maximum methylation difference (delta) between any time points after DEX treatment and baseline for each TBS amplicons. Color-shaded regions highlight the main regulatory elements in the locus. **c** Example of CpG sites showing DEX-induced methylation changes. Boxplots represent the methylation levels per time point of CpGs located in the intronic enhancers (top), proximal enhancer (bottom left), and topologically associating domain (TAD) boundaries (bottom right). Methylations of individual CpG sites are shown except for the proximal enhancer plots where the mean methylation per amplicon is shown since this region covers 94 CpG sites. *X* axes indicate the coordinate of each site or region represented. Shaded boxes indicate sites where significant DEX effects were observed at FDR ≤ 0.05 and absolute delta methylation (T_i_-baseline) ≥ 1% in at least one time point

Analysis of serum adrenocorticotropin (ACTH) and cortisol levels showed the expected suppression following DEX administration with maximal effects observed at 3 and 6 h post-treatment. In addition, DEX induced a 4.2- and 4.0-fold increase in *FKBP5* mRNA levels after 3 and 6 h of treatment, respectively, and returned to baseline level after 23 h (Fig. 1a).

#### DEX-induced transient DNAm changes—dynamics

DNAm was analyzed using our HAM-TBS technique [25], where a total of 25 amplicons covering 228 CpGs at 5 time points from baseline (0 h) to 1, 3, 6, and 23 h following DEX administration passed QC. Three amplicons located in the proximal enhancer did not pass QC due to increased CHH methylation levels (PCR18) or low coverage (PCR 28 and PCR29; < 1000 reads, Additional file 6: Table S1). DNAm analysis across the loci at baseline revealed low methylation at the TSS and higher methylation within the gene body and 3′ and 5′ flanking regions (Fig. 1b “% Methylation baseline” track).

Following an acute dose of DEX, 44 CpG sites showed significant changes in DNAm over all time points (FDR ≤ 0.05 and absolute delta methylation (T_i_-baseline) ≥ |1%|; Fig. 1b “Max. *∆* % methylation” track, shaded regions; Additional file 7: Table S2). Significant DEX-induced differential DNAm was seen as early as 1 h after treatment (*n* = 17 sites, mean absolute ∆ methylation = |2.4%|), and the largest effects were observed after 3 and 6 h (*n* = 40 sites, mean absolute ∆ methylation = |4.4%| and |5.4%|, respectively) with a range from − 17 to + 0%. Seventy-four percent of the sites, however, showed decreases in DNAm levels following DEX treatment ranging from − 17 to − 1% compared to baseline. For the majority of the sites, DNAm levels returned to baseline after 23 h of treatment while only 8 sites remained differentially methylated at FDR < 0.05 with a small change compared to baseline (mean absolute ∆ methylation = |1.8%|).

#### DEX-induced transient DNAm changes—localization

DEX-induced differentially methylated CpG sites (DMCs) were found in the proximal and intronic enhancers and co-localized with ENCODE GR binding sites and those located at the chromatin interaction blocks overlapped with ENCODE CTCF binding sites (examples are shown in Fig. 1c). Within the 82 CpGs analyzed surrounding the TSS, no DMCs were observed. Out of the 129 CpGs located in GR binding sites, 36 (28%) showed DEX-induced changes whereas only 8 (10%) out of 83 sites located in CTCF binding sites showed changes after DEX. Most of the DMCs located in GR binding sites (*n* = 30) showed reduction of DNAm following DEX (mean ∆ methylation − 3.8 ± 3.3%) with the exception of 6 sites located in the proximal enhancer (*n* = 4), intron 5 (*n* = 1) and intron 2 (*n* = 1) showing increased DNAm (mean ∆ methylation + 2.9 ± 1.3%). DMCs within CTCF binding sites located in TAD boundaries and intron 3 showed increase in DNAm (*n* = 5 sites, mean ∆ methylation + 3.6 ± 3.3%) whereas those located in the proximal enhancer showed decrease in DNAm (*n* = 3 sites; mean ∆ methylation − 3.0 ± 1.1%). Demethylated CpGs in the proximal enhancer overlap with both GR and CTCF binding sites (see Fig. 1c).

**Fig. 2:**
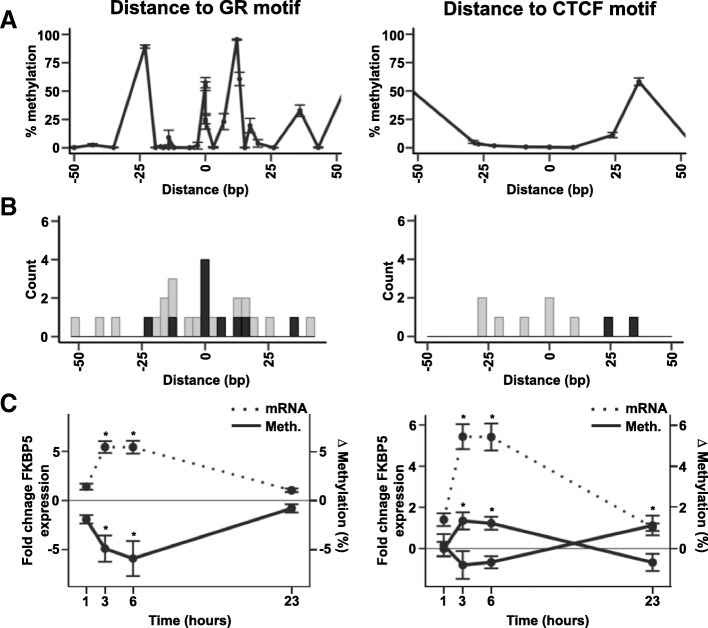
Dexamethasone (DEX)-induced DNA methylation changes (DMCs) within glucocorticoid receptor (GR) and CTCF consensus binding motifs. **a** Blood methylation levels at baseline across CpGs located within ± 50 bp of GR (left) and CTCF (right) consensus binding motifs. Line plots represent mean and SEM for each CpGs within the region. **b** Histogram representing counts of CpGs located within 50 bp of motifs (*n* = 16 for GR (left) and *n* = 9 for CTCF (right)) where DEX-induced DMC counts are shown in black. **c** Fold change *FKBP5* mRNA expression and ∆ methylation (%) in DMCs located within motifs are shown. The left panel illustrates average ∆ methylation (%) of all DMCs located within GR motifs (*n* = 4) where each of them is significant after 3 h and 6 h of DEX treatment. The right panel shows ∆ methylation (%) for the 2 DMCs located within CTCF motifs where 1 shows DEX effect after 3 h and 6 h (located at + 24 bp) and the other one only after 23 h (located at + 34 bp). Plots represent mean and SEM

To assess whether changes in DNAm might directly affect binding of GR and CTCF to DNA, we mapped the changes to their relative distance to GR and CTCF consensus binding motifs. DNAm of CpG sites within these motifs has previously shown to impair or decrease transcription factor binding [27, 28]. We used predicted DNA binding motif locations for GR and CTCF from [29] (http://compbio.mit.edu/encode-motifs/). Selecting CpGs within ± 50 bp of the consensus motif sequences (*n* = 16 for GR and *n* = 9 for CTCF), we observed that CpG sites directly in CTCF motifs consistently displayed very low DNAm levels (0.57 ± 0.10%) whereas those in NR3C1 motifs showed intermediate levels at baseline with a high variation (39.98 ± 18.43%; Fig. 2a). For CTCF motif regions, higher DNAm was observed at more distal sites at the edges of the motif. DEX-induced DMCs were found directly in GR motifs (*n* = 4) whereas none were observed within CTCF motif but at the edges of this motif (*n* = 2, Fig. 2b).

Increases in *FKBP5* mRNA levels occurred in parallel with the decrease of DNAm for 10 DMCs located within 50 bp of GR binding motifs (Fig. 2c and Additional file 6: Table S1).

### Validation of DEX-induced DNAm changes in blood in an independent sample (*n* = 89, study 2)

**Fig. 3:**
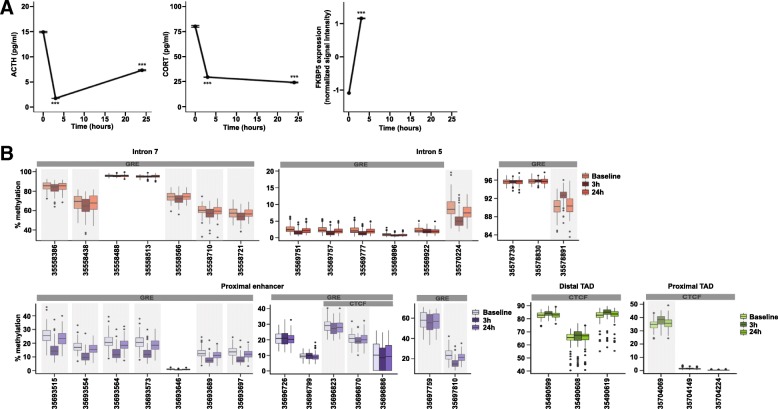
Dexamethasone (DEX)-induced transient changes in hormonal, *FKBP5* mRNA and methylation levels in an independent study. **a** Serum adrenocorticotropin (ACTH) and cortisol (CORT) levels as well as whole blood *FKBP5* mRNA levels after an oral dose of DEX in 89 subjects are shown. Peripheral blood was drawn just before administration of DEX (time = 0) as well as 3 and 20–24 h thereafter. The mean and SEM are presented for each time point. As observed in study 1, significant effect across time for ACTH (*p* value < 2.2e−16) and CORT (*p* value < 2.2e−16) levels as well as *FKBP5* mRNA levels at 3 h (*p* value < 2.2e−16) are observed. **b** Boxplots of CpG methylation levels for amplicons located in the intronic enhancers (top), proximal enhancer (bottom left), and topologically associating domain (TAD) boundaries (bottom right) showing replication of DEX-induced methylation changes. *X* axes indicate the coordinate of each site or region represented. Shaded boxes indicate sites where significant DEX effects were observed at FDR ≤ 0.05 and absolute delta methylation (T_i_-baseline) ≥ 1% in at least one time point

Using study 2, see Table 1 for demographic details, we replicated the findings in an independent sample. Similar to study 1 and has been reported previously [30], DEX treatment induced a significant decrease in CORT and ACTH levels as well as an increase in *FKBP5* mRNA levels (Fig. 3a).

Ten amplicons covering 50 CpG sites (including 25 with significant DEX effect in study 1) were selected. Of these 50 sites, 21 showed significant changes (FDR ≤ 0.05 and absolute ∆ methylation ≥ |1%|) in DNAm after DEX treatment validating 19 sites from the first study (Fig. 3b). Similar to the effects observed in the first study, DNAm changes were seen after 3 h of treatment (mean absolute ∆ methylation = |4.3%|), and for most sites (*n* = 13), DNAm returned to baseline after 24 h. Eight DMCs remained significant after 24 h of treatment but showed a much smaller effect (mean absolute ∆ methylation = |1.8%|). As observed before, the majority of sites (76%) show decrease in DNAm levels following DEX treatment with a range from − 10 to − 1% compared to baseline.

#### Inter-individual variability influencing DEX-induced methylation changes

Changes in DNA methylation in peripheral blood may reflect changes in immune cell composition. In study 2, we had data on blood cell counts (BCCs) as well as estimated immunes cell types from the Illumina 450K array data. BCCs changed over time following DEX (see Additional file 1: Figure S1A) and lymphocyte counts significantly correlated with 9 DMCs (Additional file 8: Table S3). However, when we corrected for lymphocyte counts in the models testing DEX effects on DNAm across time, all 9 sites remained significant (FDR ≤ 0.05, Additional file 8: Table S3). Since both DNAm and lymphocytes change over time with DEX, we next assessed how much of the variance in DNAm may still be explained by differences in BCCs. Comparing the standardized coefficients of lymphocyte counts change to the time changes in DNAm in a linear mixed model (LMM), we observed a significantly larger absolute coefficient at the 3-h time point (> 2.8 times larger) for all the associated sites for the changes in DNAm vs. the changes in lymphocytes (Additional file 8: Table S3 and Additional file 1: Figure S1B showing the residuals of the null model correcting for lymphocyte count). Using estimated cell types from the Illumina 450K array data, we did not observe any effects of DEX on differential cell proportions (see Additional file 1: Figure S1C). Together, these analyses suggest that change in immune cell counts with DEX are likely not a major confounder of our results. In addition to immune cell counts, we also assessed the effects of other possible confounders including sex, age, ethnicity, body mass index (BMI), smoking, and depression symptoms. We observed significant main effects of age (7 sites), sex (5 sites), smoking (6 sites), BMI (8 sites), and major depressive disorder (MDD) (4 sites) on methylation but no significant interactions with DEX (Additional file 6: Table S1 and see Additional file 2: Figure S2A–C for examples). For MDD associations with DNAm, only men were included in the analysis (*n* = 67). We have previously reported no change at baseline for FKBP5 mRNA levels between MDD patients and controls in a sub-sample of men from study 2 (29 cases and 31 controls, [30]). For the larger sample used here to test associations with MDD status in men (38 cases and 29 controls), there is also no difference in FKBP5 mRNA at baseline (*p* value > 0.05 and fold change ≥ 1.15). In contrast to the mRNA levels, out of the four FKBP5 CpG sites associated with MDD over the three time points, three sites show a difference at baseline (*p* value ≤ 0.05 and absolute delta methylation (cases-controls) ≥ |1%|) with less DNAm observed in cases (range from − 1.4 to − 2.2%, Additional file 6: Table S1).

Furthermore, we performed stepwise regression analyses using the Akaike Information Criterion (AIC) to select the main covariates influencing DNA methylation changes after DEX. This analysis was performed for the 17 CpG sites for which significant associations with DNA methylation were observed. Based on the AICs as well as the estimates of DNA methylation change after 3 h, correcting for smoking score gave the best models for all CpG sites (AIC smaller and largest variance explained). Sixteen out of seventeen sites remained significant (FDR < 0.05) after correcting for smoking (Additional file 10: Table S5). Adding the other covariates did not affect the significance of these 16 sites but increased AIC for most of them.

#### Effects of FKBP5 genotype

We and others have previously described allele-specific DNAm changes (lower methylation) in intron 7 of the *FKBP5* gene in peripheral blood cells associated with exposure to child abuse only in carriers of the minor/risk T allele of rs1360780 [11]. Therefore, we investigated whether rs1360780 genotype (CC compared to CT/TT = high mRNA induction and disease risk) had an effect on the observed significant DEX-associated methylation changes in both studies. Genotype effect on DEX-induced methylation changes over time was tested using a model testing both additive as well as interactive effects. In the first study, we observed 17 CpGs showing significant interaction (*n* = 13) or additive effects (*n* = 6) on DNAm changes over time (Additional file 6: Table S1). Despite the different timelines, 2 CpGs showed significant genotype-dependent dynamic differences in both studies, cg35558710 located in intron 7 GRE and cg35570224 located in intron 5 next to the GRE (see Additional file 3: Figure S3). We next tested whether the direction of effects in the two different genotype conformations (CC vs CT/TT) was the same in study 1 and 2. When investigating all 50 CpGs common to both studies, 27 showed the same direction of effect for genotype x DEX at time point 3 h in study 2 as compared to the effect observed overall time points in study 1, which is more than expected by chance. This was not the case for effects of genotype on DNAm differences at the time point 24 h in study 2. We then subdivided this analysis by regulatory regions. For CpGs within intronic and proximal enhancer GREs (*n* = 30), we observed a concordance of the direction of effects for genotype x time interaction between the two studies significantly more than expected chance (*p* = 0.049), but this was not the case for the 20 CpGs annotated to TADs. Overall, T carriers displayed more methylation changes over time, with differences to CC genotype carriers ranging from 5.01 to 0.01%. This analysis supports that *FKBP5* rs1360780 genotypes associate with a differential DNAm sensitivity to GR activation within GREs but not in TADs.

#### Usefulness of Illumina methylation array for assessing DEX-induced DNA methylation changes in FKBP5

Most studies investigating DNA methylation in peripheral blood in large cohorts use Illumina methylation arrays. Overlapping Illumina 450K methylation data were available at baseline and 3 h post-DEX administration in study 2 (*n* = 106 subjects, [30]) allowing to assess the extent of coverage of DEX-reactive CpGs on these arrays. The 450K array covers 56 CpGs in the *FKBP5* locus (hg19/chr6:35487554-35718452, see Additional file 4: Figure S4 and Additional file 6: Table S1 for further details) which are located mainly around the TSS (*n* = 12) and the proximal enhancer (*n* = 15) with 12 CpGs in TAD boundaries and only sparse coverage within the gene body (11 CpGs) and 3′ end (6 CpGs). None of the intronic GREs showing the biggest change in response to DEX are covered by the array. Analysis of 450K CpG sites that passed QC (52 sites) identified 13 DMCs following DEX (FDR ≤ 0.05 and absolute ∆ methylation ≥ |1%|) identifying 9 additional DMCs not covered by HAM-TBS. Overlapping sites between TBS and 450K displayed high correlation in both datasets (17 CpGs with 0.96 mean correlation) where similar DNAm changes following DEX were observed (Additional file 4: Figure S4).

#### DEX-reactive sites reside in enhancer regions with cross-tissue relevance

To understand whether the observed changes could have cross-tissue relevance, as initially shown for the GRE in intron 7 [12], we compared the chromatin state segmentations from the Roadmap Consortium (http://www.roadmapepigenomics.org/) for blood/immune cells (*n* = 29 tissues), brain cells (*n* = 10), and fibroblasts (*n* = 5 tissues) (Additional file 5: Figure S5A) for the investigated regions within the *FKBP5* locus. Common active TSS marks at the TSS and marks indicating active transcription over the gene body support the well-documented active transcription of *FKBP5* across these tissues. Focusing on the regions in *FKBP5* that showed the most prominent effects of DEX on DNAm (GRE in intron 7, intron 5, and the proximal enhancer), we observe that most of these regulatory elements show similar chromatin states, suggesting the comparable regulatory impact of these regions across these tissues (see Additional file 5: Figure S5B).

## Discussion

Here, we investigated DNAm changes in response to GR activation in the *FKBP5* locus, a gene in which DNAm changes have been shown in association with exposure to childhood trauma in both children and adults [12, 14–16]. We here show that *FKBP5* DNAm in specific enhancers is highly responsive to GR activation by DEX. We observe dynamic methylation changes over time in longitudinal samples from two independent human studies. Significant effects of DEX exposure were detected as early as 1 h following oral ingestion of DEX, with maximal effects 3–6 h later. Most changes returned to baseline within 23 h. These effects remained significant when correcting for immune cell types as well as additional covariates such as age, sex, BMI, and depression status.

The sites dynamically responsive to an acute DEX challenge in blood overlap with sites correlating with 30-day cortisol load in healthy subjects [31] as well as CpGs differentially methylated in Cushing’s syndrome patients, a disorder characterized by excess secretion of cortisol. This has been shown for CpGs within intronic GREs, especially the intron 2 and 7 GREs, for which differences were observed not only between patients with active Cushing’s syndrome and controls but also in patients with cured Cushing’s syndrome [32]. A second study using 450K data reported cg25114611, located in a GRE of the proximal enhancer, to show significantly lower methylation in patients with long-term remission of Cushing’s syndrome [33]. In our study, this site showed lower methylation following DEX in blood that remained significant after 23 h. Such overlap suggests that GR activation may—under specific circumstances—result in lasting DNAm marks. In the above studies, patients had been in remission for 7–13 years [32, 33]. Such factors contributing to more lasting changes could be the level and duration of GR activation as well as developmental timing. We have previously reported that lasting changes in DNAm following DEX treatment in hippocampal progenitor cells were observed when cells were treated during proliferation and differentiation but not when they were treated post-differentiation [12].

The DEX-responsive sites also overlap with those reported to be demethylated in both children and adults exposed to early trauma [11]. Although these sites do not show durable demethylation after 23 h, the overlap suggests that these adversity-related epigenetic changes in *FKBP5* GREs could be mediated via GR-dependent effects through prolonged exposure to GCs, as observed in children exposed to trauma [13].

Our results give new insight into the previously described allele-specific methylation changes of intron 7 GRE CpGs that may contribute to the *FKBP5* x early trauma associations with risk for a number of psychiatric disorders ([10], for review). Several studies report that lower methylation of *FKBP5* GRE intron 7 is more pronounced in child abuse-exposed individuals carrying haplotypes tagged by the T allele of rs1360780 [12, 15, 34]. This functional allele has been associated with increased transcriptional activation of *FKBP5* by GR [12, 35]. Here, we show that healthy individuals carrying this allele have a different dynamic of DNAm changes following GR activation in GREs of *FKBP5*. Such a difference in epigenetic dynamics may also contribute to the fact that environmental risk factors linked to stress hormone activation, such as early adversity, could have more lasting effects in individuals carrying this specific allele. Such allele-specific differences in the dynamics of DNAm change may relate to the reported allele-specific differences in gene transcription. As illustrated in Fig. 2c, dynamic DNAm changes in *FKBP5* GREs inversely correlate with changes in RNA expression.

As expected from the literature, methylation levels of CTCF binding sites at the *FKBP5* locus were low (0.57 ± 0.10%). In general, it has been shown that CTCF occupancy is inversely correlated with DNA methylation and that DNA methylation at CpGs located directly in the core binding motif can inhibit CTCF binding [27, 36–40]. A gain of methylation directly at CTCF binding sites can lead to loss of CTCF binding and therefore disruption of chromatin interactions which can lead to a dysregulated gene expression [37]. DEX-induced DMCs were not found directly at the core motif of the CTCF binding sites but at close distance and show small changes (< |2%|, Fig. 2B). The upstream and downstream flanking regions around the CTCF core motif have been implicated in influencing CTCF binding stability [41]. However, how such a small change in DNA methylation at these flanking regions influences CTCF occupancy has not yet been experimentally addressed so that their exact role remains unknown. Overall, our data suggests that the DEX-induced methylation effects concentrate on GR and not CTCF binding sites. These changes in DNA methylation may thus alter enhancer function (as shown in reporter gene assays [12]) but will not likely result in more profound 3D chromatin changes, such as loop disruptions.

In addition to genetically induced altered dynamics of DEX responsivity, other factors may also contribute to long-term effects on *FKBP5* methylation in the context of early adversity. Factors associated with child abuse such as smoking, BMI, and depression all had main effects on *FKBP5* methylation, including in dynamically responsive sites (see Additional file 6: Table S1 and Additional file 2: Figure S2). In addition, age also had a main effect on some CpGs. The limited age range of our cohorts, however, prohibits to analyze the influence of age, including childhood and adolescence, in more depth. Whether such differences in baseline methylation contribute to the long-term effect of early adversity on *FKBP5* methylation needs to be investigated in more detail in longitudinal cohorts. A limitation of this study is the lack of information on early or more recent adversity. Future studies will need to address the influence of these environmental factors on GR-induced DNA methylation dynamics.

Overall, the effects of DEX on *FKBP5* methylation were mostly in the direction of lower methylation following GR activation. In fact, the observation that DEX can induce DNA methylation changes is not unique to the *FKBP5* locus and has been observed at different genomic loci [42]. Several mechanisms could contribute to differences in DNAm. One is a change in cell type composition, favoring cell types with no methylation at these sites within mixed tissues. While this is a possible explanation for the changes observed, the fact that these effects withstand correction for changes in cell composition over time (see Additional file 1: Figure S1B and Additional file 8: Table S3) and that DEX has no significant effect on specific immune cell types estimated from the genome-wide DNAm data suggests that at least some of these effects likely happen within specific cells. Re-assessing our results in sorted cells would give more information into which cell types or cell characteristics are associated with the highest epigenetic reactivity in humans. A study in mice, using genome-wide bisulfite sequencing after cell sorting, observed GC-induced methylation changes primarily in blood T cells [43]. On the other hand, mapping of enhancers across many tissues, including different immune cells (see Additional file 5: Figure S5), suggests that most of the GR-responsive enhancers exert a shared function and may thus show similar epigenetic responses to GR activation across different cell types and tissues.

A reduction in DNA methylation following GR activation could also be mediated via a transcription factor binding-mediated DNA demethylation which has been reported for GR binding to GREs [5]. The mechanisms for this targeted DNA demethylation are not fully understood, but mechanisms involving DNA repair have been proposed [9]. Similar to the GR-induced transient changes observed here in blood, rapid cyclical pattern of DNAm in response to estrogen stimulation in breast cancer cells has been reported. The ERα-responsive gene *pS2* undergoes rapid demethylation and remethylation cycles following activation of transcription with estrogen [44]. The authors [44] implicated a coordinated binding of DNA methyltransferases, glycosylase, and base excision repair proteins in these processes. The process of demethylation of the pS2 promoter investigated in the above paper is thought to involve Dnmt3a/b that is able to deaminate 5mC. The resulting abasic site (AP site) of this deamination is subsequently repaired by recruiting p68, TDG, and BER proteins (AP endonuclease, DNA polymerase β, and DNA ligase I). The rapid GR-induced demethylation followed by remethylation within 23 h observed here in blood cells may occur via similar mechanisms given the reported kinetics of this enzymatic process. In addition, when aggregating data from our study for all GREs and mapping DNAm changes to the distance from the consensus GR binding site, we observe high levels of methylation within the consensus binding site and these central CpGs are also the ones with dynamic reduction following GR activation (see Fig. 2a). These observations would support GRE-centric active DNA demethylation. The mechanisms that would then associate with the more lasting changes in remitted Cushing’s patients and in individuals exposed to childhood trauma could relate to different actions of DNA-methyl binding proteins such as MeCP2 and polycomb complexes that would interfere with DNA-driven demethylation/remethylation [45].

## Conclusions

Taken together, these data provide novel insight into possible mechanisms of stress and trauma-related changes in DNAm and gene x stress interactions, suggesting a role of GR-dependent methylation changes at least for a subset of the effects. These effects are best investigated using targeted approaches, such as HAM-TBS [25], as most of the reactive enhancer CpGs are not covered on the current Illumina arrays. The observed dynamics of these changes in peripheral blood have consequences on epigenetic association studies in humans, where controlling for cortisol plasma levels appears to be an important factor. Given that dynamic changes in DNAm that can be induced by a single dose of DEX and given their overlapping sites correlating with 30-day cortisol load as well as with lasting changes observed in patients with Cushing’s syndrome, critical questions arise for the long-term epigenetic consequences of the therapeutic use of GCs. Additional research in larger samples, with different exposure lengths and intensity, different tissues, and different developmental stages, will be necessary to better understand this phenomenon on a genome-wide and organism-wide level. Cataloging the moderation of these GR-induced epigenetic effects by common gene variants may further help in identifying genes contributing to risk and resilience to stress-related psychiatric disorders.

## Methods

### Study samples

#### Study 1

Healthy male participants (*n* = 26, age 25.4 ± 2.9) were given 1.5 mg of DEX orally at 12:00, see [46] for more details on the study samples and [30, 47–50] for the choice of dose. Peripheral blood was drawn just before administration of DEX as well as 1, 3, 6, and 23 h thereafter. Nineteen of the 26 samples were selected for TBS for a balanced rs1360780 genotype distribution (7 subjects with TT genotype, 6 with TC, and 6 with the CC genotype).

#### Study 2

The second sample consisted of 89 Caucasian subjects and were also exposed to 1.5 mg of DEX orally as previously described in [42]. Here, DEX was administered at 6 pm and blood draws occurred immediately before the dose of DEX and then 3 hours as well as ~ 18 hours (from 17.5 to 21 h) later. The subset comprised of 30 healthy probands (female = 1; male = 29) and 59 inpatients with depressive disorders (female = 21; male = 38) with an age of 41.64 ± 13.96 (mean age ± SD).

The demographics of both studies are reported in Table 1.

### DNA and RNA extraction of study samples

For both studies, DNA was extracted from frozen EDTA blood using the Elmer Chemagic 360 Instrument (PerkinElmer chemagen Technologie GmbH, Baesweiler, Germany) in combination with the chemagic DNA Blood Kit special 400 (PerkinElmer chemagen Technologie GmbH, Baesweiler, Germany). Thirty-three blood samples of study 2 were only collected in PAXgene tubes (PreAnalytiX GmbH, Hombrechtikon, Switzerland) for the ~ 18 h time point. For these samples, DNA was extracted from PAXgene tubes using PAXgene Blood DNA Kit (QIAGEN GmbH, Hilden, Germany). Blood for RNA was stored in PAXgene tubes, and RNA was extracted using the PAXgene Blood RNA Kit (QIAGEN GmbH, Hilden, Germany). All samples had an RNA integrity number of at least 7.0.

### Genotyping

#### Study 1

All participants were genotyped for the rs1360780 allele using hybridization probes (forward primer: CCTTATTCTATAGCTGCAAGTCCC, reverse primer: TCTGAATATTACCAGGATGCTGAG, rs1360780_LC: Red640-AAATTCTTACTTGCTACTGCTGGCACAAGAGA-Phosphate, rs1360780_FL: CAGAAGGCTTTCACATAAGCAAAGTTACACAAAAC-Fluorescein). Genomic DNA was amplified using the LightCycler 480 Genotyping Master mix (Roche, Mannheim, Germany) with the following cycling conditions: 95 °C, 10 s; 45× (95 °C, 1 s; 56 °C, 10 s; 72 °C, 10 s); 95 °C, 1 min; and 40 °C, 1 min, ramped up to 85 °C using a ramp rate of 0.57 °C/s and one acquisition per °C on a LightCycler480 II Instrument (Roche, Mannheim, Germany).

#### Study 2

Genotyping for this cohort is described in [47] and was based on Illumina 660K genotyping arrays.

### Assessment of endocrine and immune measures

Cortisol and ACTH levels were assessed as described in [48, 51]. For the measurement of plasma cortisol concentrations, a radioimmunoassay kit was used (INC Biomedicals, Carlson, CA). Plasma ACTH concentrations were assessed by automated electrochemiluminescence immunoassay using Roche Cobas immunoassay analyzer (Roche, Basel, Switzerland). In the second study, additionally, plasma DEX levels were measured at the 3-h and 20–24-h time point using mass spectrometry as described in [48] and differential blood cell counts evaluated at all three time points as reported in [51].

### Gene expression analysis via quantitative real-time PCR

#### Study 1

*FKBP5* mRNA expression levels in blood samples of the first study were assessed as follows. The generation of cDNA was achieved using SuperScript™ II reverse transcriptase (Thermo Scientific Inc., Schwerte, Germany). Subsequently, the cDNA was amplified in duplicates in a LightCycler 480 Instrument II (Roche, Mannheim, Germany) using the LightCycler 480 SYBR Green I Master kit (Roche, Mannheim, Germany) and primer spanning Exon 10-11 (forward primer: AAAAGGCCAAGGAGCACAAC, reverse primer: TTGAGGAGGGGCCGAGTTC; cycling conditions: 95 °C, 10 s; 45× (95°,10 s; 58 °C, 10 s; 72 °C, 10 s)). C_t_ values were used to calculate relative expression levels according to [52] normalized on *YWHAZ* expression (Universal ProbeLibrary probe #77; cycling conditions according to the manufacturer’s recommendations; Roche, Mannheim, Germany) for normalization and mean assay efficiencies.

#### Study 2

RNA expression from study 2 was done using Illumina HT12v4 arrays previously described in [48]. In this study, blood RNA samples were only available for two time points (baseline and 3 h after DEX exposure).

### Assessment of DNA methylation in study samples

DNA methylation levels in both studies were analyzed using the HAM-TBS approach, comprising an optimized PCR panel of 28 amplicons in the *FKBP5* locus [25]. These data were complemented by Illumina 450K methylation arrays. An overview of the methylation data obtained can be found in Additional file 6: Table S1.

#### Study 1

HAM-TBS [25] on the *FKBP5* locus was run for all 28 amplicons on DNA from each blood sample (baseline, 1 h, 3 h, 6 h, and 23 h post-DEX administration) in a single sequencing run with 302 CpG sites analyzed.

#### Study 2

*FKBP5* locus DNAm levels were assessed using both HAM-TBS [25] (10 amplicons covering 50 CpGs from each blood sample (baseline, 3 h and 18 h post-DEX administration)) and 450K methylation arrays (56 CpGs at baseline and 3 h post-DEX administration).

### Targeted bisulfite sequencing of the *FKBP5* locus

This method has been described in detail in [25] and offers good performance of amplicon bisulfite sequencing assays in a technology comparison by the Blueprint consortium [53].

#### Amplicon selection and amplification by PCR

We optimized the amplifications of 28 regions covering 302 CpGs within GR and/or CTCF binding sites as well as the transcription start site of the *FKBP5* locus (see Additional file 9: Table S4 for primers and mapping of the amplicons). In order to reduce cost and maximize the number of samples per sequencing run, triplicate bisulfite treatments were performed for each sample and then pooled to run one PCR amplification per amplicon [25]. Overall, 200 ng to 500 ng of DNA was used per sample and bisulfite treated using the EZ DNA Methylation Kit (Zymo Research, Irvine, CA). Twenty nanograms of bisulfite-converted DNA was then used for each PCR amplification employing Takara EpiTaq HS Polymerase (Clontech, Saint-Germain-en-Laye, France) and 49 amplification cycles. PCR amplicons were then quantified with the Agilent 2200 TapeStation (Agilent Technologies, Waldbronn, Germany) and pooled in equimolar quantities for each sample. AMPure XP beads (Beckman Coulter, Krefeld, Germany) were used for a double size selection (200–500 bp) to remove primer dimers and high molecular DNA fragments.

#### Sequencing

Libraries were generated using the TruSeq DNA PCR-Free HT Library Prep Kit (Illumina, San Diego, CA) according to the manufacturer’s instructions. Each library was quantified with the Qubit® 1.0 (Thermo Fisher Scientific Inc., Schwerte, Germany), normalized to 4 nM and pooled. Library concentration and fragment sizes were checked via Agilent’s 2100 Bioanalyzer (Agilent Technologies, Waldbronn, Germany) and quantitative PCR using the Kapa HIFI Library quantification kit (Kapa Biosystems, Wilmington, MA). Paired-end sequencing was performed on an Illumina MiSeq Instrument (Illumina, San Diego, CA) with their MiSeq Reagent Kit v3 (2× 300 cycles) with the addition of 30% of PhiX Library.

#### Sequencing data processing

The quality of the sequencing reads was checked with FastQC (http://www.bioinformatics.babraham.ac.uk/projects/fastqc), and Illumina adapter sequences were removed using Cutadapt v.1.9.1. Bismark v.0.15.0 was used for the alignment to a restricted reference limited to our PCR targets. In order to stitch paired-end reads, an in-house Perl script has been developed to remove the low-quality ends of the paired-end reads if they overlapped. The methylation levels for all CpGs, CHGs, and CHHs were quantified using the R package methylKit. The resulting DNAm calls were submitted to a 3-step quality control. First, PCR artifacts introducing false CpGs of low coverage at 0 or 100% methylation level were removed. Second, CHH methylation levels were analyzed, and samples with insufficient bisulfite conversion rate (< 95%) were removed. Finally, CpG sites with a coverage lower than 1000 reads were excluded.

### Illumina 450K methylation arrays

#### Study 2

Illumina 450K arrays were processed as described in [42]. Smoking scores were predicted from DNAm data as described in [54] as this information was not available for all subjects. Blood cell ratios were estimated from the DNAm data using the Houseman algorithm [55]. Normalized beta values of 52 CpG sites located within the *FKBP5* locus (hg19/chr6:35,487,554-35,718,452) were extracted from the 425,883 probes that passed quality control (QC).

### Statistical analysis

#### DEX effects in study 1 and study 2

Linear mixed models (LMMs) were used to assess the effects of DEX treatment over time on either ACTH, cortisol, *FKBP5* expression, or DNAm levels for each CpG sites in both studies. All models were run adjusting for intra-individual variability as random effect using the “lmer” function of the Lme4 package in R [56]. *p* values were calculated using the Wald chi-square test from the Car package [57]. False discovery rate (FDR) was applied to correct for multiple testing on methylation *p* values. Post hoc analysis comparing each time point to baseline was ran using LMMs for each site showing significant results from above (FDR ≤ 0.05) to determine at which time point the effect was observed. Differentially methylated CpG sites (DMCs) were called when FDR from the mixed model was ≤ 0.05 and absolute mean methylation differences between significant time points and baseline (*p* value ≤ 0.05) were ≥ 1%.

The power to detect significant DNAm changes after DEX administration was performed using the function “powerSim” from the R package SIMR [56] with 100 simulations for each CpG sites profiled in both studies. These analyses used a significance (alpha) level of 0.05 and minimum effect of absolute methylation difference ≥ 1% between 3 or 6 h and baseline for study 1 (delta T_3_–T_0_ and T_6_–T_0_) and 3 hours and baseline for study 2 (delta T_3_–T_0_). In our discovery sample (study 1), an average power of 96.6% (bootstrap 95% CI = (94.7, 97.9)) overall the 228 CpG sites was predicted to detect a minimum difference of 1% in methylation after 3 and/or 6 h of DEX administration. Over all the 50 CpG sites profiled in the replication cohort (study 2), an average power of 88.8% (bootstrap 95% CI = (81.2, 93.4)) was predicted to detect a minimum difference of 1% in methylation after 3 h of DEX administration. These results indicate that both cohorts have sufficient power (> 80%) to detect a minimum difference of 1% in methylation after DEX administration with our repeated measures design (5 sampling times in study 1 and 3 in study 2). Although the power in both studies is sufficient to detect a 1% change in methylation, much of the effect observed was larger than 3% (for 66% and 62% of the total significant sites in study 1 and 2, respectively).

In addition, parametric bootstrap using the “bootMer” function of the “lme4 package” in R using 100 simulations, for the mixed models of the 50 CpG sites profiled in study 2, was performed. The bootstrap results including the measures of bias and standard error as well as confidence intervals are given in Additional file 11: Table S6. This analysis revealed that the results are stable as the 95% confidence intervals from the 3-h and 24-h time points indicate a change in DNA methylation for the all sites identified with LMM (FDR ≤ 0.05 and absolute delta methylation ≥ 1%).

#### Inter-individual factors influencing DEX-induced DNAm changes

To assess inter-individual factors influencing the observed changes in DNAm following DEX administration, each CpG site showing significant DEX effects in study 2 was tested (*n* = 21 CpGs). LMMs were used to assess the association between DNAm change over time and blood cell counts, age, sex, smoking, BMI, and MDD status for each CpG sites. All models were run adjusting for intra-individual variability as random effect using the “lmer” function of the Lme4 package in R [56]. *p* values were calculated using the Wald chi-square test from the Car package [57]. Stepwise regression analysis was also performed on 17 sites showing association with either age, sex, smoking, BMI, or MDD status to select the main confounding variables influencing DNAm change over time. AICs and the DNAm estimates at 3 h of these models were used to select the best model (Additional file 10: Table S5).

*FKBP5* genotype effect on DEX-induced methylation changes over time was first assessed on 44 CpG sites in study 1 and 21 sites in study 2 showing DNAm changes at any time point post-administration of DEX. LMMs were used as described above. Methylation for each CpG was regressed against the main effect of time (DEX) and rs1360780 risk allele (CC or CT/TT) with and without the interaction term of genotype * time point while adjusting for intra-individual variability. *p* values of the additive and interaction effects for each time points were calculated using the Wald chi-square test.

We assessed if the direction of effects was concordant across studies based on the binomial distribution. Assuming that a CpG site shows the same direction of effect in both studies by chance with a probability of 0.5, we determined the probability to observe the present or even a higher number of CpG sites with concordant directions.

## Additional files

Additional file 1: **Figure S1.** Change in blood cell counts after DEX administration. A) Actual blood cell counts at baseline and after DEX administration for granulocytes, monocytes, and lymphocytes in 54 subjects from study 2. B) Boxplot of DNAm residuals from a null model correcting for associated variance in lymphocyte counts across time in 54 subjects from study 2. Post hoc analysis correcting for lymphocyte counts revealed significant change in DNAm after 3 h of DEX for all sites (*p* value < 0.1e−18). C) Predicted blood cell proportions from 450K methylation data in study 2 using the Houseman algorithm [55]. (PDF 260 kb)

Additional file 2: **Figure S2.** DEX-induced changes in DNAm are also influenced by factors associated with early life adversity. Examples of three CpG sites were significant associations with fixed factors including age, sex, BMI, smoking score, and major depression were observed. (PDF 150 kb)

Additional file 3: **Figure S3.** CpG sites with significant genotype-dependent dynamic methylation differences in both studies. Effects of rs1360780 genotype on DEX-induced DNA methylation changes in 2 sites located in intron 7 and 5 enhancers. The % methylation levels for rs1360780 risk allele carriers CT/TT and CC carriers following DEX exposure are shown for each study. Methylation of CpG 35558710 shows significant interaction effect at 23 h in study 1 (*Χ*^2^ = 5.69, *p* value = 0.02) and additive effect at 3 h in study 2 (*Χ*^2^ = 4.15, *p* value = 0.04) with risk allele genotype. Significant interactions between risk allele genotype and DEX on methylation were observed for CpG 35570224 at 6 h and 23 h post-treatment in study 1 (*Χ*^2^ = 7.59, *p* value = 0.006 and *Χ*^2^ = 6.0, *p* value = 0.01) and at 24 h in study 2 (*Χ*^2^ = 4.36, *p* value = 0.04). Points and error bars represent mean and SEM for each genotype. (PDF 101 kb)

Additional file 4: **Figure S4.** Replication of dexamethasone (DEX)-induced methylation changes (*n* = 106 subjects) analyzed by Illumina 450K arrays. A) Genome browser shot illustrating the location of TBS amplicons assessed as well as the location of the 450K Illumina probes within the *FKBP5* locus (hg19/chr6:35487554-35718452). *CTCF-ChIA-PET -*track indicating the locations of CTCF factor mediated chromatin interactions determined by Chromatin Interaction Analysis with Paired-End Tag (ChIA-PET) data extracted from lymphoblastoid cell line (GM12878, [26]). Chromatin interactions are represented by PET blocks connected with an horizontal line; *CTCF-ChIP-seq and GR-Chip-seq—*regions of transcription factor binding derived from chromatin immunoprecipitation (ChIP) experiments in multiple cell lines from the ENCODE project; *blood TBS amplicons*—locations of targeted bisulfite sequencing (TBS) amplicons assessed in blood of study 1; *450K probe locations*—locations of Illumina probes from the 450K array. B) Boxplot of DNAm levels using TBS or Illumina 450K approach for the overlapping CpG sites showing methylation changes after DEX using TBS. *p* values of linear mixed models for each time point compared to baseline or vehicle are indicated as follows: *≤ 0.05, **≤ 0.01, ***≤ 0.001. Note that although cg125114611 show significant DEX effect using 450K array, this site has a methylation change after DEX of − 0.4% which did not reach our threshold of |1%|. (PDF 480 kb)

Additional file 5: **Figure S5.** Comparison of chromatin states in *FKBP5* across brain, immune/blood, and fibroblasts. A) Genome browser shot illustrating the chromatin states of the *FKBP5* locus (hg19 / chr6:35487554-35718452) across brain, immune/blood, and fibroblasts. *FKBP5 splicing variants—*visualization of two splicing variants of *FKBP5*; *TBS amplicons*—locations of targeted bisulfite sequencing (TBS) amplicons; *450K probe locations*—locations of Illumina probes from the 450K array; *CTCF-ChIP-seq* and *GR-Chip-seq*—regions of transcription factor binding derived from chromatin immunoprecipitation (ChIP) experiments in multiple cell lines from the ENCODE project; *CTCF-ChIA-PET* and *PolII-ChIA-PET—*track indicating the locations of CTCF factor or PolII mediated chromatin interactions determined by Chromatin Interaction Analysis with Paired-End Tag (ChIA-PET) data extracted from lymphoblastoid cell line (GM12878, [26]). Chromatin interactions are represented by PET blocks connected with an horizontal line; *Ensembl Reg Build—*overview of the Ensembl regulatory build which represents an annotation of regions likely to be involved in gene regulation; *ChroHMM—*this track displays the chromatin state segmentation of the *FKBP5* locus for selected brain, immune/blood, and fibroblast cells from the Roadmap Consortium. The primary core marks segmentation has been used which visualize predicted functional elements as 15 states, which are displayed at the bottom of the figure. B) Quantification of the 15 chromatin states at key regulatory regions (transcription start site (TSS), topologically associating domains (TAD), proximal Enhancer (proxE), and intronic GREs (iGRE)) of the *FKBP5* locus. Chromatin states were averaged over brain (*n* = 10), immune/blood (*n* = 29), and fibroblasts (*n* = 5) cells. (PDF 390 kb)

Additional file 6: **Table S1.** Details on the CpG sites assessed in *FKBP5* locus and summary of the results obtained using HAM-TBS and Illumina 450K array in both studies. (XLSX 78 kb)

Additional file 7: **Table S2.** Summary statistic from linear mixed models testing the change in methylation after DEX for each CpG sites assessed in study 1 (*n* = 228) including post-hoc analysis for each time point. In bold are sites with significant DEX-induced methylation change. (XLSX 234 kb)

Additional file 8: **Table S3.** Summary statistic from linear mixed models testing the change in methylation after DEX including lymphocyte cells counts as covariate for each CpG sites associated with change in lymphocyte counts (*n* = 9) in study 2. (XLSX 50 kb)

Additional file 9: **Table S4.** Location of HAM-TBS amplicons and primer sequences used to analyze *FKBP5* CpGs. (XLSX 15 kb)

Additional file 10: **Table S5.** Results from stepwise regression analyses comparing the LMM models without covariate, with smoking score alone, or with all the associated covariates performed on 17 CpG sites showing association with either age, sex, smoking, BMI, or MDD status in study 2. (XLSX 35 kb)

Additional file 11: **Table S6.** Summary statistic from the linear mixed models testing the change in methylation after DEX for 50 CpG sites profiled in study 2 as well as the measures of bias, standard error, and confidence intervals using bootstraps with 100 simulations. (XLSX 46 kb)

## Abbreviations

4-OHT: 4-Hydroxytamoxifen
BCCs: Blood cell counts
BER: Base excision repair
ChIA-PET: Chromatin Interaction Analysis with Paired-End Tag
ChIP: Chromatin immunoprecipitation
CpGs: Cytosine guanine dinucleotides
CTCF: CCCTC-binding factor
DEX: Dexamethasone
DMC: Differentially methylated CpG site
DNAm: DNA methylation
DNMTs: DNA methyltransferases
ELS: Early-life stress
GC: Glucocorticoid
GR: Glucocorticoid receptor
GRE: Glucocorticoid response element
HAM-TBS: High-accuracy methylation measurements via targeted sisulfite sequencing
HPC: Hippocampal progenitor cell
HWE: Hardy-Weinberg equilibrium
LMM: Linear mixed model
MAF: Minor allele frequency
MDD: Major depressive disorder
PBMCs: Peripheral blood mononuclear cells
PcG: Polycomb complexes
SVA: Surrogate variable analysis
SVs: Surrogate variables
TAD: Topologically associating domains
TETs: Ten-eleven translocation methylcytosine dioxygenase proteins
TSS: The transcription start site
