# Supplementary figures and images for: Identification of dynamic glucocorticoid-induced methylation changes at the FKBP5 locus
Source: Clin Epigenetics. 2019 May 23;11:83. doi: 10.1186/s13148-019-0682-5 (PMC6533766; doi:10.1186/s13148-019-0682-5)

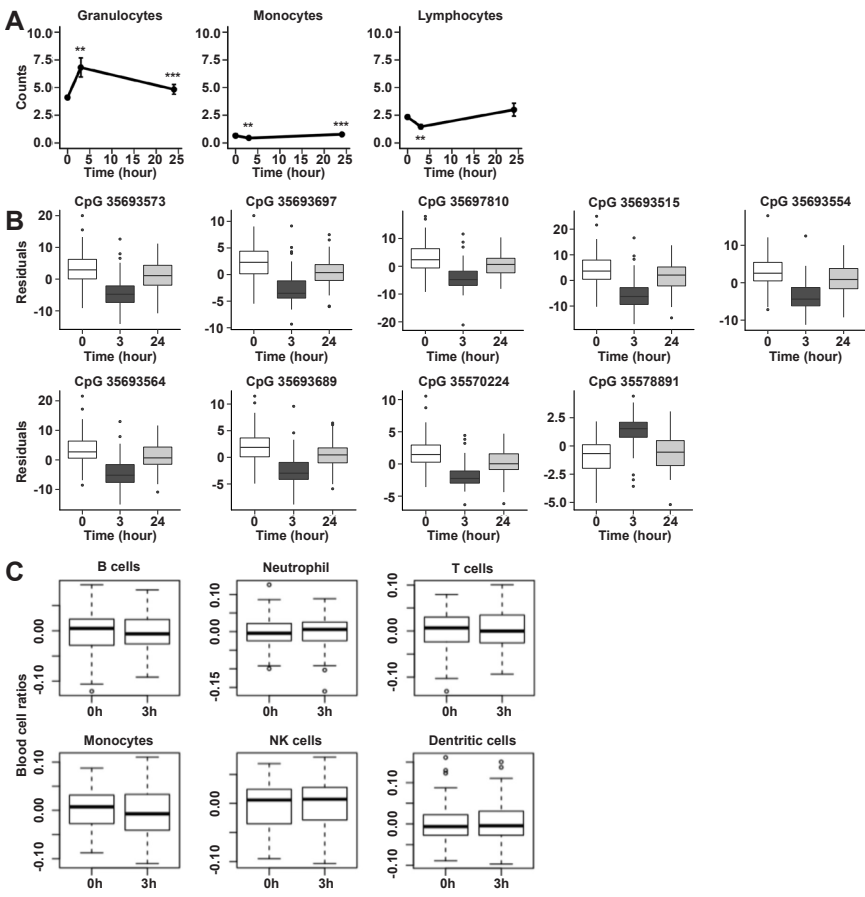

Supplement: Supplementary file 1 — Figure S1. Change in blood cell counts after DEX administration. A) Actual blood cell counts at baseline and after DEX administration for granulocytes, monocytes, and lymphocytes in 54 subjects from study 2. B) Boxplot of DNAm residuals from a null model correcting for associated variance in lymphocyte counts across time in 54 subjects from study 2. Post hoc analysis correcting for lymphocyte counts revealed significant change in DNAm after 3 h of DEX for all sites (p value < 0.1e−18). C) Predicted blood cell proportions from 450K methylation data in study 2 using the Houseman algorithm [55]. (PDF 260 kb) [file 13148_2019_682_MOESM1_ESM.pdf]

**A**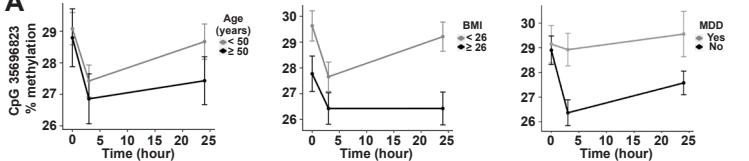**B**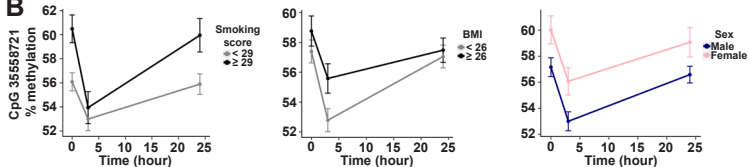**C**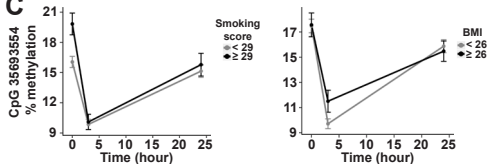

Supplement: Supplementary file 2 — Figure S2. DEX-induced changes in DNAm are also influenced by factors associated with early life adversity. Examples of three CpG sites were significant associations with fixed factors including age, sex, BMI, smoking score, and major depression were observed. (PDF 150 kb) [file 13148_2019_682_MOESM2_ESM.pdf]

Intron 7  
CpG 35558710

Intron 5  
CpG 35570224

GRE

Study 1

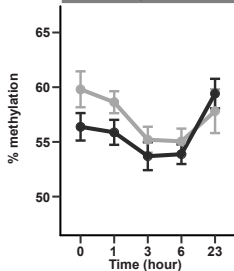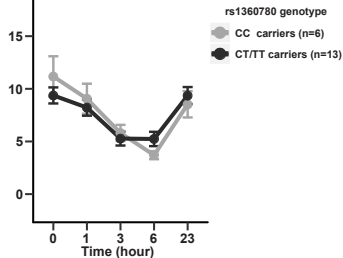

Study 2

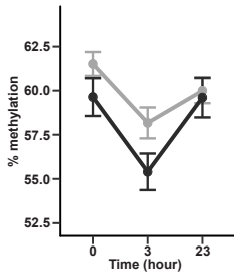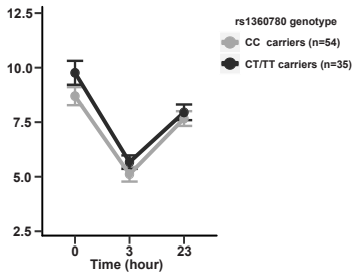

Supplement: Supplementary file 3 — Figure S3. CpG sites with significant genotype-dependent dynamic methylation differences in both studies. Effects of rs1360780 genotype on DEX-induced DNA methylation changes in 2 sites located in intron 7 and 5 enhancers. The % methylation levels for rs1360780 risk allele carriers CT/TT and CC carriers following DEX exposure are shown for each study. Methylation of CpG 35558710 shows significant interaction effect at 23 h in study 1 (Χ2 = 5.69, p value = 0.02) and additive effect at 3 h in study 2 (Χ2 = 4.15, p value = 0.04) with risk allele genotype. Significant interactions between risk allele genotype and DEX on methylation were observed for CpG 35570224 at 6 h and 23 h post-treatment in study 1 (Χ2 = 7.59, p value = 0.006 and Χ2 = 6.0, p value = 0.01) and at 24 h in study 2 (Χ2 = 4.36, p value = 0.04). Points and error bars represent mean and SEM for each genotype. (PDF 101 kb) [file 13148_2019_682_MOESM3_ESM.pdf]

**A**

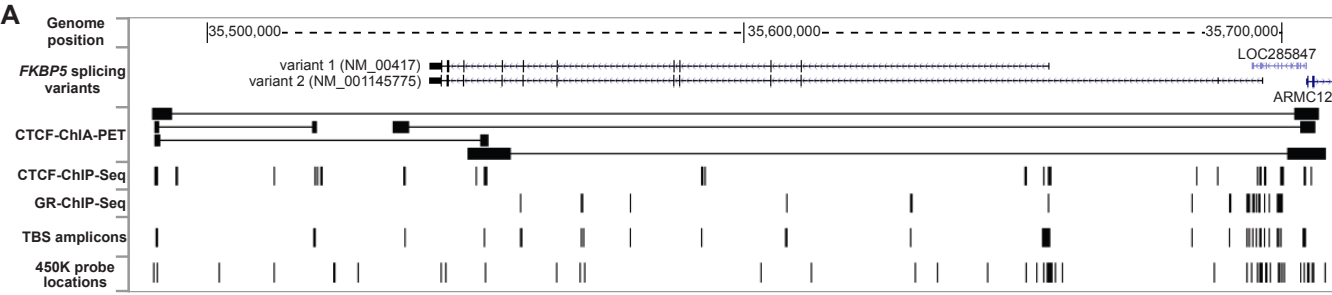

# B

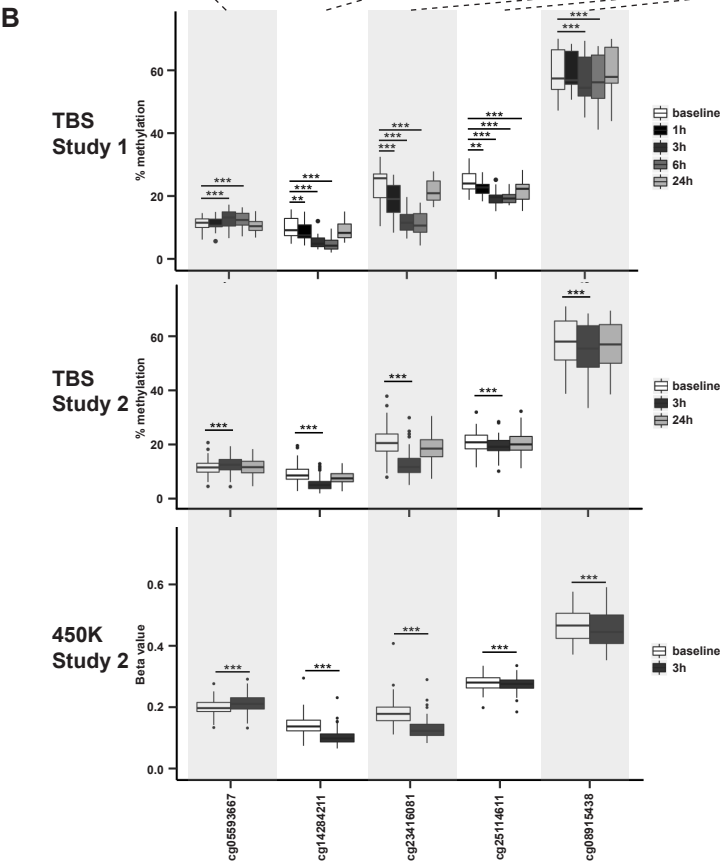

Supplement: Supplementary file 4 — Figure S4. Replication of dexamethasone (DEX)-induced methylation changes (n = 106 subjects) analyzed by Illumina 450K arrays. A) Genome browser shot illustrating the location of TBS amplicons assessed as well as the location of the 450K Illumina probes within the FKBP5 locus (hg19/chr6:35487554-35718452). CTCF-ChIA-PET -track indicating the locations of CTCF factor mediated chromatin interactions determined by Chromatin Interaction Analysis with Paired-End Tag (ChIA-PET) data extracted from lymphoblastoid cell line (GM12878, [26]). Chromatin interactions are represented by PET blocks connected with an horizontal line; CTCF-ChIP-seq and GR-Chip-seq—regions of transcription factor binding derived from chromatin immunoprecipitation (ChIP) experiments in multiple cell lines from the ENCODE project; blood TBS amplicons—locations of targeted bisulfite sequencing (TBS) amplicons assessed in blood of study 1; 450K probe locations—locations of Illumina probes from the 450K array. B) Boxplot of DNAm levels using TBS or Illumina 450K approach for the overlapping CpG sites showing methylation changes after DEX using TBS. p values of linear mixed models for each time point compared to baseline or vehicle are indicated as follows: *≤ 0.05, **≤ 0.01, ***≤ 0.001. Note that although cg125114611 show significant DEX effect using 450K array, this site has a methylation change after DEX of − 0.4% which did not reach our threshold of |1%|. (PDF 480 kb) [file 13148_2019_682_MOESM4_ESM.pdf]
