# Supplementary material for: Identification of dynamic glucocorticoid-induced methylation changes at the FKBP5 locus
Source: Clin Epigenetics. 2019 May 23;11:83. doi: 10.1186/s13148-019-0682-5 (PMC6533766; doi:10.1186/s13148-019-0682-5)
Supplement: Supplementary file 5 — Figure S5. Comparison of chromatin states in FKBP5 across brain, immune/blood, and fibroblasts. A) Genome browser shot illustrating the chromatin states of the FKBP5 locus (hg19 / chr6:35487554-35718452) across brain, immune/blood, and fibroblasts. FKBP5 splicing variants—visualization of two splicing variants of FKBP5; TBS amplicons—locations of targeted bisulfite sequencing (TBS) amplicons; 450K probe locations—locations of Illumina probes from the 450K array; CTCF-ChIP-seq and GR-Chip-seq—regions of transcription factor binding derived from chromatin immunoprecipitation (ChIP) experiments in multiple cell lines from the ENCODE project; CTCF-ChIA-PET and PolII-ChIA-PET—track indicating the locations of CTCF factor or PolII mediated chromatin interactions determined by Chromatin Interaction Analysis with Paired-End Tag (ChIA-PET) data extracted from lymphoblastoid cell line (GM12878, [26]). Chromatin interactions are represented by PET blocks connected with an horizontal line; Ensembl Reg Build—overview of the Ensembl regulatory build which represents an annotation of regions likely to be involved in gene regulation; ChroHMM—this track displays the chromatin state segmentation of the FKBP5 locus for selected brain, immune/blood, and fibroblast cells from the Roadmap Consortium. The primary core marks segmentation has been used which visualize predicted functional elements as 15 states, which are displayed at the bottom of the figure. B) Quantification of the 15 chromatin states at key regulatory regions (transcription start site (TSS), topologically associating domains (TAD), proximal Enhancer (proxE), and intronic GREs (iGRE)) of the FKBP5 locus. Chromatin states were averaged over brain (n = 10), immune/blood (n = 29), and fibroblasts (n = 5) cells. (PDF 390 kb) [file 13148_2019_682_MOESM5_ESM.pdf]

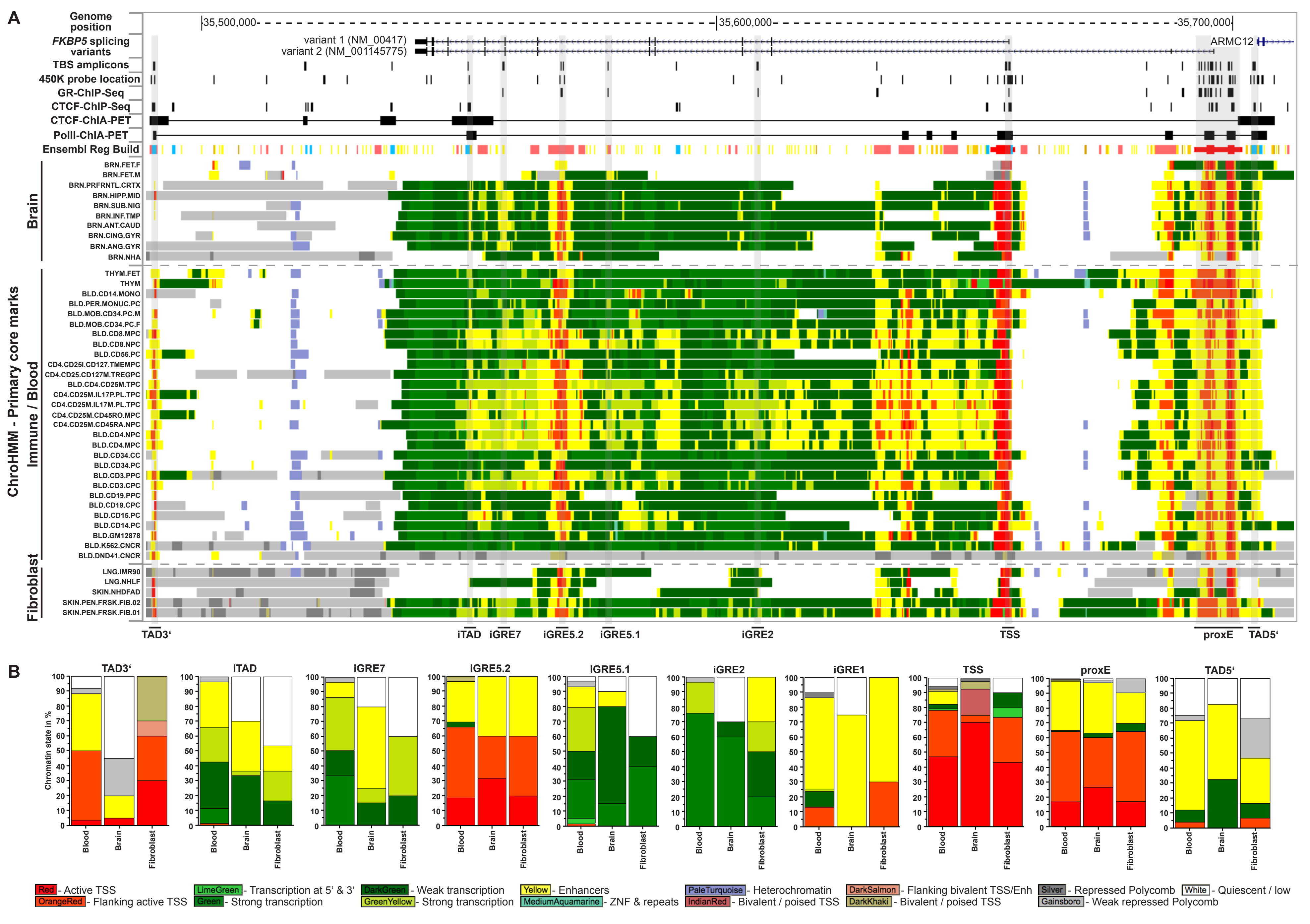

A
Genome position
35,500,000
35,600,000
35,700,000
FKBP5 splicing variants
variant 1 (NM_00417)
variant 2 (NM_001145775)
ARMC12
TBS amplicons
450K probe location
GR-ChIP-Seq
CTCF-ChIP-Seq
CTCF-ChIA-PET
PolII-ChIA-PET
Ensembl Reg Build
ChroHMM - Primary core marks
Brain
BRN.FET.F
BRN.FET.M
BRN.PRFRNTL.CRTX
BRN.HIPP.MID
BRN.SUB.NIG
BRN.INF.TMP
BRN.ANT.CAUD
BRN.CING.GYR
BRN.ANG.GYR
BRN.NHA
Immune / Blood
THYM.FET
THYM
BLD.CD14.MONO
BLD.PER.MONUC.PC
BLD.MOB.CD34.PC.M
BLD.MOB.CD34.PC.F
BLD.CD8.MPC
BLD.CD8.NPC
BLD.CD56.PC
CD4.CD25I.CD127.TMEMPC
CD4.CD25.CD127M.TREGPC
BLD.CD4.CD25M.TPC
CD4.CD25M.IL17P.PL.TPC
CD4.CD25M.IL17M.PL.TPC
CD4.CD25M.CD45RO.MPC
CD4.CD25M.CD45RA.NPC
BLD.CD4.NPC
BLD.CD4.MPC
BLD.CD34.CC
BLD.CD34.PC
BLD.CD3.PPC
BLD.CD3.CPC
BLD.CD19.PPC
BLD.CD19.CPC
BLD.CD15.PC
BLD.CD14.PC
BLD.GM12878
BLD.K562.CNCR
BLD.DND41.CNCR
Fibroblast
LNG.IMR90
LNG.NHLF
SKIN.NHDFAD
SKIN.PEN.FRSK.FIB.02
SKIN.PEN.FRSK.FIB.01
TAD3‘
iTAD
iGRE7
iGRE5.2
iGRE5.1
iGRE2
TSS
proxE
TAD5‘
B
Chromatin state in %
TAD3‘
iTAD
iGRE7
iGRE5.2
iGRE5.1
iGRE2
iGRE1
TSS
proxE
TAD5‘
0
10
20
30
40
50
60
70
80
90
100
Blood
Brain
Fibroblast
Red - Active TSS
OrangeRed - Flanking active TSS
LimeGreen - Transcription at 5‘ & 3‘
Green - Strong transcription
DarkGreen - Weak transcription
GreenYellow - Strong transcription
Yellow - Enhancers
MediumAquamarine - ZNF & repeats
PaleTurquoise - Heterochromatin
IndianRed - Bivalent / poised TSS
DarkSalmon - Flanking bivalent TSS/Enh
DarkKhaki - Bivalent / poised TSS
Silver - Repressed Polycomb
Gainsboro - Weak repressed Polycomb
White - Quiescent / low
